# Research Progress of Biomarkers for Immune Checkpoint Inhibitors on Digestive System Cancers

**DOI:** 10.3389/fimmu.2022.810539

**Published:** 2022-04-13

**Authors:** Jingting Wang, Xiao Ma, Zhongjun Ma, Yan Ma, Jing Wang, Bangwei Cao

**Affiliations:** ^1^ Department of Oncology, Beijing Friendship Hospital, Capital Medical University, Beijing, China; ^2^ Department of Comprehensive Medicine, Beijing Shijingshan Hospital, Beijing, China

**Keywords:** immune check inhibitor (ICI), digestive system cancers, immunotherapy, predict therapeutic effectiveness, biomarker

## Abstract

Immunotherapy represented by immune checkpoint inhibitors has gradually entered a new era of precision medicine. In view of the limited clinical benefits of immunotherapy in patients with digestive system cancers, as well as the side-effects and high treatment costs, development of biomarkers to predict the efficacy of immune therapy is a key imperative. In this article, we review the available evidence of the value of microsatellite mismatch repair, tumor mutation burden, specific mutated genes or pathways, PD-L1 expression, immune-related adverse reactions, blood biomarkers, and patient-related biomarkers in predicting the efficacy of immunotherapy against digestive system cancers. Establishment of dynamic personalized prediction models based on multiple biomarkers is a promising area for future research.

## 1 Introduction

**Figure 1:**
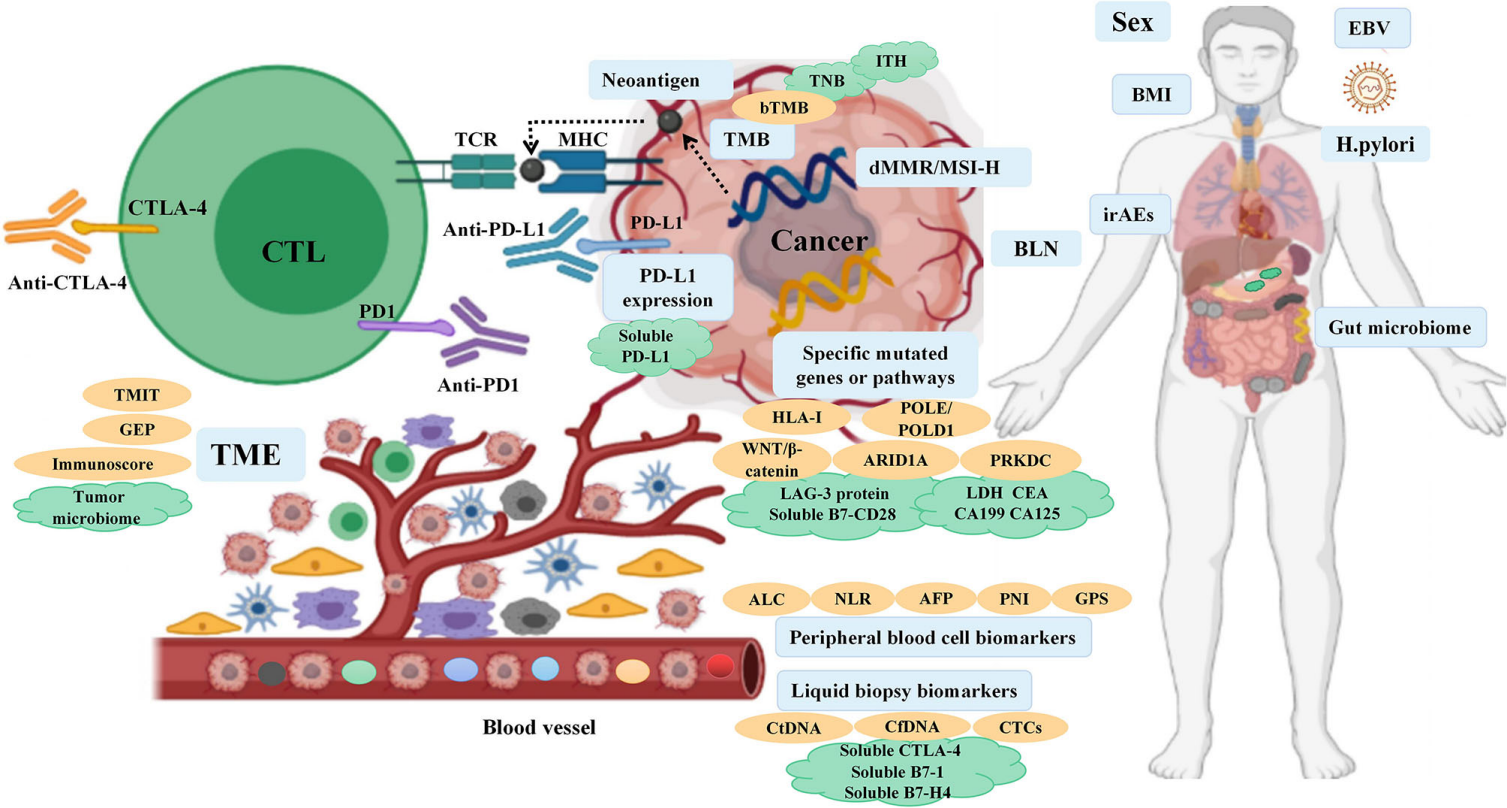
Overview of the biomarkers for predicting the response to ICI therapy in digestive system cancers.

Immune checkpoint inhibitor (ICI) therapy has transformed the treatment landscape for advanced-stage forms of many cancers, especially non-small cell lung cancer (NSCLC), melanoma, head and neck squamous cell carcinoma, kidney cancer and digestive system cancers. Despite several studies showing good long-term outcomes of these therapies, in clinical practice, the overall response rate (RR) in patients undergoing ICI treatment is unsatisfactory due to the heterogeneity of tumors, with only 20%–40% of patients benefiting from it in most scenarios. Therefore, identification of predictive biomarkers that can help screen patients who are most likely to respond to immunotherapy will help reduce unnecessary treatment costs and avoid immune-related adverse events (irAEs). In the era of personalized medicine, a variety of immunohistochemical techniques and high-throughput sequencing of the human genome are poised to play an increasingly important role by identifying clinically-relevant biomarkers using specimens such as blood samples and tissue specimens. In the context of cancer treatment, assessment of these biomarkers at baseline and at different time-points during treatment can provide valuable information to guide therapeutic decision-making. Moreover, use of a combination of clinical and molecular biomarkers is likely to play an important role in clinical decision-making. Extensive research has been conducted on biomarkers of immunotherapy efficacy in the context of NSCLC and melanoma, but there is a paucity of related studies on digestive system cancers. In this review, we discuss the currently available biomarkers that can help predict the efficacy of ICI therapy in patients with digestive system cancers (**Figure 1**).

## 2 Biomarkers of ICI Therapeutic Efficacy Against Digestive System Cancers

### 2.1 Tumor Genome Biomarkers

#### 2.1.1 Mismatch Repair Deficiency and Microsatellite Instability

Microsatellites are short tandem repeats throughout the human genome characterized by single nucleotide, dinucleotide or high nucleotide repetitions, and the number of repetitions is 10–50 times. Compared with normal cells, tumor cells exhibit altered length of microsatellites due to the insertion or deletion of repeat units, a phenomenon referred to as microsatellite instability (MSI). Mismatch repair (MMR) expression loss can cause accumulation of mismatches during DNA replication, leading to the occurrence of MSI. In 2017, the United States Food and Drug Administration (FDA) approved PD-1 antibody drugs for the treatment of "mismatch-repair-deficient (dMMR)/microsatellite instability-high (MSI-H)" type solid tumors. This is the first anti-tumor therapy that is not based on the source of the tumor, but on molecular biomarkers in a wide range of tumors, laying the foundation for tumor ICI treatment markers. The reported incidence of MSI-H in gastric cancer (GC) and colorectal cancer (CRC) is 9% and 15%–20%, respectively, while the incidence is as high as 69.5% in Lynch syndrome adenocarcinoma (1–3). Clinically, MSI has been used as an important prognostic molecular biomarker in patients with CRC and other solid tumors, and has been used to inform formulation of adjuvant treatment plans; it has also been used to assist in the screening of Lynch syndrome.

In the landmark CHECKMATE 142 trial, MSI-H/dMMR patients with metastatic CRC showed a high degree of benefit from nivolumab treatment with an objective response rate (ORR) of 31%, of which 51 patients (69%) had disease control for ≥12 weeks, and all patients survived during the 12-month follow-up period (10). These patients showed a more sustained clinical benefit from nivolumab and ipilimumab dual immunotherapy, with ORR as high as 55% (11). In the study by Le et al. (9), ORR (40% vs 0%) and 20-week progression-free survival (PFS) rate (78% vs 11%) of metastatic CRC patients treated with pembrolizumab were significantly greater than those of proficient mismatch repair (pMMR) patients. The median progression-free survival (mPFS) and median overall survival (mOS) were not reached in the cohort with dMMR CRC, but were 2.2 and 5.0 months, respectively, in the cohort with pMMR CRC at a median follow-up period of 36 weeks (9). Subsequently, Le have expanded this study to evaluate efficacy of PD-1 blockade in patients with advanced dMMR cancers across 12 different tumor types. Objective radiographic response was observed in 53% and complete response (CR) was achieved in 21% of patients. Neither mPFS nor mOS has been reached yet over a median follow-up time of 12.5 months (2). The results of KEYNOTE-164 and KEYNOTE-158 also further demonstrated the sustained clinical benefit of pembrolizumab in patients with MSI-H/dMMR metastatic CRC (13, 14). In the study by Noor et al., although the MSI-H/dMMR phenotype accounted for only 0.8% of pancreatic cancer patients, in the 7 patients treated with ICI, the mPFS was 8.2 months, and the mOS was not reached during the follow-up period of 6.8 months, resulting in better clinical benefit (12).

dMMR tumors often have a higher density of tumor infiltrating lymphocytes (TIL), and the "high immunogenicity" established by the large number of mutant neoantigens in dMMR tumors may be closely related to the efficacy of PD-1 inhibitors (9). The current National Comprehensive Cancer Network (NCCN) guidelines recommend the use of immunohistochemical staining methods to detect MSI from the protein level, and molecular-level polymerase chain reaction methods to detect specific microsatellite repeat sequence amplification to determine MSI status; however, there are still problems in the detection or interpretation process. With the widespread application of high-throughput sequencing platforms, next-generation gene sequencing (NGS) is being gradually applied for detection of microsatellite status, and can greatly increase the sensitivity of detection (18).

#### 2.1.2 Tumor Mutation Burden

Tumor mutation burden (TMB) is an exploration of tumors at the level of human genome. TMB is defined as the number of somatic mutations in the whole genome after including the germ line DNA variants. The enhanced tumor immunogenicity and low immunosuppressive tumor micro-environment (TME) of high tumor mutation burden (TMB-H) can affect the sensitivity and clinical efficacy of ICI therapy based on the underlying assumption that TMB-H can create antigenic peptides (15, 19). In 2020, based on the findings from the phase 2 KEYNOTE-158, TMB was approved by FDA for the treatment of patients with any unresectable or metastatic non-dMMR/MSI TMB-H (TMB≥10 mut/MB) solid cancer that has progressed on prior therapy and for which no alternative treatment options are available. The KEYNOTE-158 study included patients with advanced malignancies involving the anal canal and biliary system; after a median follow-up of 37.1 months, objective response (OR) were observed in 30 (29%) of 102 patients in the tissue TMB-H (tTMB-H) group (≥10 mut/MB) and 43 (6%) of 688 patients in the non tTMB-H group. These findings indicated that the tTMB-H subgroup of patients may show a robust tumor response to pembrolizumab monotherapy (7). In a retrospective study of 1638 tumor patients, including CRC and hepatocellular carcinoma (HCC), who received immunotherapy and underwent full genetic testing and TMB assessment, TMB-H was independently related to efficacy of immunotherapy. TMB-H patients (TMB≥20 mut/MB) had significantly higher RR (58% vs 20%) and mPFS (12.8 vs 3.3 months) than those with low tumor mutation burden (TMB-L), and there was a positive linear correlation between TMB-H and the efficacy of anti-PD-1/PD-L1 monotherapy (4). In a study of 58 patients with advanced GC treated with toripalimab, TMB-H patients (TMB≥12 mut/MB) had significantly longer OS than patients with TMB-L (14.6 vs 4 months) (5). Furthermore, in the study by Schrock et al., the mPFS for TMB-H patients with metastatic CRC has not been reached (median follow-up >18 months) while the mPFS of TMB-L patients was 2 months (6). According to a recent research, TMB-H CRC in the Cancer Genome Atlas showed a trend towards increased RR and significantly improved prognosis (15). The above research results suggest that TMB is a novel and useful biomarker in patients with MSI-H mCRC, GC, and HCC, which can help stratify patients based on the likelihood of clinical benefit of ICI therapy.

However, the FDA approval for use of TMB has been met with mixed reviews. McGrail's analysis determined the correlation between TMB and survival benefit by analyzing genetic data of more than 10,000 cancer patients. The results failed to support the use of TMB-H as a biomarker for ICI treatment in all solid cancer types. TMB-H was found to predict the efficacy of immunotherapy for category I cancers types (such as CRC) where neoantigen load is related to CD8 T cell levels, while TMB-H was not found to predict response in category II cancer types where neoantigen load is not positively correlated with CD8 T-cell levels. Therefore, as the predictive value of TMB differs in different tumor types, further tumor type-specific research is necessary (15).

There is no clear consensus about several aspects of the use of TMB in predicting the efficacy of ICI therapy, such as the definition of the TMB-H threshold. In addition, whether TMB is a predictive or prognostic marker, or both, is not clear. Wu found that TMB has different effects on survival outcomes in different cancer types and can be incorporated in prognostic and risk stratification (20). Another important shortcoming is that TMB cannot more specifically reflect the immunogenicity of neoantigens. As an emerging auxiliary indicator of the TMB, the tumor neoantigen burden (TNB) is an indicator that reflects the total number of neoantigens in tumor cells. Tumor neoantigens can be presented by human MHC molecules and activate immune cells, and patients with more neoantigens are more likely to continue to benefit from immunotherapy. There is a highly significant correlation between TNB and anti-PD-1 treatment response, and TNB can directly and more accurately predict the response of anti-PD-1 treatment than TMB (21). TNB predicts the benefit of immunotherapy for digestive system cancers. Further research is required to explore whether TNB can replace TMB as a valuable immune predictor. In summary, the somatic mutation rate of tumors and the potential to form neoantigens are related to their sensitivity to ICI therapy. NGS technology used in combination with MSI and TMB analysis may be a more accurate tool for selecting cancer patients for immunotherapy (22).

In clinical settings, tissue biopsy is the standard for cancer diagnosis and treatment, and tTMB test is the primary choice when tumor tissue can be obtained or is adequate for use. However, TMB measurement in tumor tissue biopsy specimen is typically limited to a specific region of the tumor and may not accurately reflect the mutation panorama of the entire tumor; in particular, it may not capture the spatial and temporal heterogeneity found in patients with metastasis, and the main reason for the failure of detection lies in insufficient tumor tissue and/or tumor cells. Recent studies have emphasized that detecting blood TMB (bTMB) offers practical advantages over use of tissue to detect tTMB. It is a simple and non-invasive alternative to tissue biopsy. It has advantages of fast turnaround time, high patient compliance, good specificity, low heterogeneity, and repeated sampling. Yang evaluated the bTMB of peripheral blood circulating tumor DNA (ctDNA) in patients with esophageal cancer, and found that the RR in the high bTMB group (bTMB >8) was significantly greater than that in the low bTMB group (bTMB ≤8) (61.5% vs 47.1%), confirming the feasibility of bTMB as an immunotherapy biomarker for esophageal cancer (8). In the MYSTIC study, bTMB was found to have a higher detection success rate than tissue samples (81% vs 63%) (23). However, the limitations of bTMB are that detection of mutations in plasma samples is influenced by the amount of tumor shedding, the depth of coverage, and clonal hematopoietic mutations. In addition, ctDNA content may also affect bTMB prediction efficiency. In order to overcome this limitation, in a study of lung cancer, ctDNA content of patients was incorporated into the algorithm model of bTMB, and the concept of low allele frequency bTMB was proposed, suggesting a significant correlation between ctDNA content and survival (24). It was more accurate than traditional bTMB in predicting the benefits of immunotherapy in this population. This study indicates the need to explore the potential biological mechanism of the predictive value of bTMB and to further optimize the predictive value of bTMB in digestive system tumors. 

Intratumoral heterogeneity (ITH) refers to spatial or temporal heterogeneity with respect to the distribution of genomic diversity in a single tumor, resulting from cumulative gene mutations. Patients with low ITH were found to perform better in presentation and recognition of neoantigens during immunotherapy, predicting the prognosis in NSCLC (25). The research indicated that the response to immunotherapy can be optimally predicted by using the combination of ITH and TMB, and subsequently verified a consistent role of ITH in esophageal and GC. Further studies are required to expand the use of ITH in predicting the response of digestive system cancers to immunotherapy.

The level of correlation between subsets of biomarkers should also be noted, in particular, mutation metrics, such as subclonal TMB and cloned TMB. Kevin collated whole-exome and transcriptomic data from >1000 patients with 8 cancer types (including CRC) who were treated with ICI, to validate the multivariate predictors of immunotherapy (26). They found that clonal TMB was the strongest predictor of ICI response, followed by TMB and CXCL9 expression, while subclonal TMB and somatic copy alteration burden showed no significant predictive ability. They also observed a negative association between the burden of subclonal mutations and all indicators of immune infiltration, such as characteristics of CD8 effects, which is consistent with the recent emphasis on immunosuppressive effects of high burden of subclonal mutations. Tumors with high levels of neoantigens have a lower antigen dose than homogenous tumors with a high clonal neoantigen load, thus reducing the chances of recognizing T cells that respond to subclonal neoantigens (27). When T cells respond to subclonal neoantigens, these cells will not be able to target all tumor cells, thus limiting the attack on the tumor as a whole (28). These studies highlighted that neoantigen heterogeneity may influence immune surveillance and support the use of clonal neoantigens as biomarkers for predicting the efficacy of immunotherapy.

#### 2.1.3 Specific Mutated Genes or Pathways

The NGS technology provides reliable targets and biomarkers of response to ICI treatment of digestive system cancers. The available data provides novel insights for defining biomarker-driven immunotherapy responses in specific genes mutations.

In the study by Harding, activation of altered Wnt/β-catenin signaling in HCC patients was found to be associated with lower disease control rate (DCR) (53% vs 0), shorter mPFS (7.4 vs 2.0 months) and mOS (15.2 vs 9.1 months) (16). In HCC, Wnt/CTNNB1 mutations characterize the immune excluded class and WNT activation leads to T-lymphocyte exclusion, making it a predictive biomarker of intrinsic innate resistance to ICI therapy in HCC (29). Therefore, HCC patients with alterations of non-WNT pathway notably respond or derive clinical benefit from immunotherapy. However, due to the small sample size and confounding factors in this study, further large-scale studies are required to confirm the clinical significance of Wnt/CTNNB1 mutations. In addition, *POLE* and *POLD1* are genes encoding DNA polymerase subunits that play a key role in the proofreading fidelity of DNA replication. *POLE* mutations are more common in patients with right colon cancer, stable microsatellites, and young men. In a study of 47,721 patients with various cancers including CRC, esophagogastric cancer, cholangiocarcinoma, HCC, and pancreatic ductal adenocarcinoma (PDAC), patients with *POLE* or *POLD1* mutations were found to have significantly higher TMB than those without these mutations, and their OS was significantly longer than that of wild-type populations (34 vs 18 months), which has been verified as an independent predictor of ICI treatment (17). Furthermore, Hu collectively reviewed the association between *ARID1A* inactivation and MMR, TMB, PD-L1, and TME. They found that *ARID1A* mutation may potentially serve as a predictive biomarker for ICI therapy in GC (30). The potential basis for *ARID1A* deficiency and immunotherapeutic sensitivity may be related to its disruption of mismatch repair, promotion of tumor mutation, increase of PD-L1 expression, and regulation of TME. In addition, the TMB of *PRKDC* mutation samples was significantly higher than that of *PRKDC* wild-type samples, especially in GC and CRC. Based on the TCGA tumor database, the expressions of CD8+T cells, NK cells, immune checkpoints, and chemokines were significantly increased in *PRKDC* mutation samples. *PRKDC* mutations predict favorable response to ICI therapy in lung cancer and melanoma, and can be further promoted in gastrointestinal (GI) cancers in the future (31). Moreover, human leukocyte antigen (*HLA*) gene is a polymorphic region in human genome. Increase in somatic mutation rate of *HLA* gene is significantly correlated with *HLA* dysfunction, which is a potential mechanism of immune escape, involved in carcinogenesis and tumor progression, and affects the efficacy of immunotherapy. *HLA* class I genotype polymorphism was shown to be associated with better prognosis in patients with NSCLC and advanced melanoma (32). In patients with advanced esophageal cancer, the immunotherapy RR (85.71% vs 27.27%) and mPFS (7.683 vs 1.867 months) of patients with *HLA* heterozygous type were significantly higher and longer than those of homozygous type, respectively, suggesting that *HLA* typing may be a potential biomarker for predicting immunotherapy efficacy (see **Table 1**) (8).

**Table 1:** Predictive ability of tumor genome-related biomarkers for response to ICI therapy for digestive system cancers.

Type of predictors		Cancer type	ICI therapy	Number	Outcome	TMB-H	TMB-L	Reference
**TMB**	tTMB	Cancer (including CRC, HCC)	anti-PD-1/PD-L1	151	RR	58%	20%	Goodman 2017 (4)
					PFS	12.8 months	3.3 months	
					OS	Not reached	16.3 months	
		Chemo-refractory AGC	anti-PD-1 (Toripalimab)	58	OS	14.6 months	4 months	Wang 2019 (5)
		MSI-H mCRC	anti-PD-1/PD-L1	22	mPFS	Not reached	2 months	Schrock 2019 (6)
		Advanced solid tumors (including anal and biliary)	anti-PD-1 (Pembrolizumab)	790	ORR	29%	6%	Marabelle 2020 (7)
	bTMB	Advanced esophageal cancer	anti-PD-1	30	RR	61.5%	47.1%	Yang 2019 (8)
**MMR/MSI status**						**dMMR**	**pMMR**	
		mCRC	anti-PD-1 (Pembrolizumab)	41	ORR	40%	0%	Le 2015 (9)
					20-week PFS rate	78%	11%	
					ORR	40%	0%	
					DCR	90%	11%	
					mPFS	Not reached	2.2 months	
					mOS	Not reached	5.0 months	
		MSI-H mCRC	anti-PD-1 (Nivolumab)	74	ORR	31%	–	Overman 2017 (10)
		MSI-H mCRC	Dual immunotherapy (Nivolumab plus Ipilimumab)	119	ORR	55%	–	Overman 2018 (11)
		PDAC	ICI therapy	7	mPFS	8.2	–	Noor 2021 (12)
					mDOR	Not reached	–	
		12 different cancers (including CRC/GEA/pancreas/small intestine/cholangiocarcinoma)	anti-PD-1 (Pembrolizumab)	86	CR	21%	–	Le 2017 (2)
		Treatment-refractory, MSI-H/dMMR mCRC	anti-PD-1 (Pembrolizumab)	128	ORR	33%	–	Le 2019 (13)
					mDOR	Not reached	–	
		MSI-H/ dMMR cancers (including GC, cholangiocarcinoma, and pancreatic cancers)	anti-PD-1 (Pembrolizumab)	233	ORR	34.3%	–	Marabelle 2019 (14)
					mPFS	4.1 months	–	
					mOS	23.5 months	–	
		category I cancer types (including CRC)	anti-PD-1/PD-L1/ CTLA-4	>10,000	ORR	39.8%	4.1%	McGrail 2021 (15)
						**Gene mutation**	**Gene wild**	
**Gene mutation predictors**	WNT/β-catenin pathway	HCC	anti-PD-1/PD-L1/ CTLA-4	31	DCR	0%	53%	Harding 2019 (16)
					PFS	2.0 months	7.4 months	
					OS	9.1 months	15.2 months	
	POLE/POLD1 mutations	Multiple cancer (including CRC, esophagogastric cancer)	anti-PD-1/PD-L1/ CTLA-4	47,721	OS	34 months	18 months	Wang F 2019 (17)
	HLA class I genotype (heterozygote/homozygous type)	Advanced esophageal cancer	anti-PD-1	25	RR	85.71%	27.27%	Yang 2019 (8)
					mPFS	7.683 months	1.867 months	

AGC, advanced gastric cancer; CRC, colorectal cancer; HCC, hepatocellular carcinoma; TMB, tumor mutation burden; MMR, mismatch-repair; MSI, microsatellite instability; mCRC, metastatic colorectal cancer; DCR, disease control rate; PFS, progression-free survival; mOS, median overall survival; OS, overall survival; mPFS, median progression-free survival; mDOR, median disease control rate; ORR, objective response rate; RR, response rate; PDAC, pancreatic ductal carcinoma; RR, response rate.

Confirmation of the above preliminary data on the predictive effect of gene mutations in current and future clinical trials will widen the prospects of their use to inform treatment decision-making with respect to immunotherapy for digestive system cancers.

### 2.2 PD-L1 Expression

PD-L1, expressed on immune and tumor cells, interacts with PD-1 on immune checkpoint proteins that negatively regulate anti-tumor immune response, which enables tumor cells to evade immune surveillance. PD-L1 expression is closely related to a wide pattern of coregulated gene expression including T cell activation markers, T cell cytokine recruitment, and antigen presentation across multiple cell types (33). The expression level of PD-L1 seems to reflect the balance between host immune response and cancer immune escape, and it is higher in malignant tumor tissues than in precancerous lesions and normal tissues (34, 35). The combined positive score (CPS), tumor proportion score (TPS), and immune cell proportion score (IPS) are commonly used clinical evaluation criteria for PD-L1 expression, as a direct predictor of anti-PD-L1/PD-1 immunotherapy efficacy.

Several large randomized controlled trials have demonstrated the potential use of PD-L1 expression as a predictive biomarker (36, 38). In the KEYNOTE-059 study, 259 patients with previously treated advanced-stage GC or gastro-esophageal junction adenocarcinomas received pembrolizumab; the ORR was 15.5% in patients with a CPS of ≥1 versus 6.4% in those with a CPS of <1 (36). In the KEYNOTE-062 trial, among patients with advanced GC who received pembrolizumab as first-line treatment, those with CPS ≥ 10 showed longer OS (17.4 vs 10.6 months) than those with CPS ≥ 1 (38). Moreover, in heavily pretreated patients with advanced, metastatic adenocarcinoma (AC) or squamous cell carcinoma (SCC) of the esophagus, those with PD- L1 CPS ≥10 showed significantly greater ORR compared to those with CPS <10 (13.8% vs 6.3%) (41). For second-line treatment of AC or SCC, in the Chinese subgroup of the KEYNOTE-181 study, the mOS of those with PD-L1 CPS ≥ 10 was nearly two-fold greater than those with PD-L1 CPS <10 (12.0 vs 6.4 months) (37).

However, there is no clear consensus on the predictive ability of PD-L1 expression as a marker of response to ICI therapy. In the cohort of toripalimab monotherapy for advanced refractory GC, mPFS (5.5 vs 1.9 months, *P* = 0.092) and mOS (12.1 vs 5.3 months, *P* = 0.45) were highly increased in PD-L1 positive patients, but the differences in survival outcomes were not statistically significant (5). Furthermore, exploratory biomarker analysis of PD-L1 expression (≥1% or <1%) showed no significant difference in ORR (28.6% vs 27.7%) and DCR ≥12 weeks (52.4% vs 74.5%) in the nivolumab arm for CRC patients, suggesting that PD-L1 is not a predictive biomarker in these patients (10). Patients with dMMR/MSI-H mCRC showed response to nivolumab plus ipilimumab dual immunotherapy, irrespective of tumor PD-L1 expression (11). A meta-analysis of 9 studies found that high PD-L1 expression rate is associated with poor prognosis of ICI therapy for PDAC (43). In the CHECKMATE 040 study, baseline PD-L1 status in tumor cells showed no apparent effect on ORR in patients with advanced HCC treated with nivolumab (TPS≥1% 26% vs TPS<1% 19%) (40). Use of PD-L1 expression in combination with other biomarkers seems to reduce the offset of a single marker and predict ICI therapy efficacy more accurately. Chemo-refractory GC patients who were TMB-H and PD-L1 positive showed long-term benefits of toripalimab with respect to ORR (33.3% vs 3.0%), PFS (2.7 vs 1.9 months), and OS (12.1 vs 4 months) compared with those with TMB-L and PD-L1 negative status (5).

However, for PD-L1 expression assessment assays, CPS and TPS may have different values for predicting survival benefits. Compared with TPS, CPS is not limited to PD-L1 expression in tumor cells, but includes the sum of all PD- L1 positive cells (tumor cells, lymphocytes, and macrophages). In the KEYNOTE 224 trial, PD-L1 expression assessed by CPS score showed a correlation with the ORR benefit of pembrolizumab treatment in HCC patients (CPS≥1 32% vs CPS<1 20%); however, there was no significant correlation between TPS and therapeutic efficacy, suggesting that the combination of CPS score and TPS score may improve the predictive value of PD-L1 immunohistochemical assay (**Table 2**) (39). Moreover, the use of CPS to determine PD-L1 expression appears to be a more sensitive prognostic biomarker than TPS in GC, but this conclusion has not been generalized to all digestive system cancers (44).

**Table 2:** Predictive ability of PD-L1 expression for response to ICI therapy for digestive system cancers.

Cancer type	ICI therapy	Number	Assessment assay	Outcome	PD-L1 positive		PD-L1 negative	Reference
GC, AEG	anti-PD-1 (Pembrolizumab)	259	CPS	ORR	15.5%		6.4%	Fuchs 2018 (36)
				CRR	2.0%		2.8%	
EAC, ESCC	anti-PD-1 (Pembrolizumab)	123	CPS	OS	12 months		6.4 months	Chen 2019 (37)
Advanced GC	anti-PD-1 (Pembrolizumab)	763	CPS	OS	17.4 months		10.6 months	Shitara 2020 (38)
Chemotherapy refractory GC	anti-PD-1 (Toripalimab)	55	TPS	ORR	37.5%		8.5%	Wang 2019 (5)
				PFS	5.5 months		1.9 months	
				OS	12.1 months		5.3 months	
MSI-H mCRC	anti-PD-1 (Nivolumab)	68	TPS	ORR	28.6%		27.7%	Overman 2017 (10)
				DCR for ≥12 weeks	52.4%		74.5%	
MSI-H mCRC	Dual immunotherapy (Nivolumab plus Ipilimumab)	119	TPS	ORR	54%		52%	Overman 2018 (11)
				DCR	77%		78%	
Advanced HCC previously treated with Sorafenib	anti-PD-1 (Pembrolizumab)	104	CPS	ORR	32%		20%	Zhu 2018 (39)
			TPS		43%		22%	
Advanced HCC	anti-PD-1 (Pembrolizumab)	174	TPS	ORR	26%		19%	El-Khoueiry 2017 (40)
Pretreated advanced, metastatic adenocarcinoma or ESCC	anti-PD-1 (Pembrolizumab)	121	CPS	ORR	13.8%		6.3%	Shah 2018 (41)
					PD-L1 mRNA**^High^**		PD-L1 mRNA **^Low^**	
MSS CRC	anti-PD-1	210	PD-L1 mRNA expression	mOS	Not reached		60 months	Liu 2021 (42)
**Combined predictor**								
						**PD-L1 positive** **+TMB-H**	**PD-L1 negative** **+TMB-L**	
Chemotherapy refractory GC	anti-PD-1 (Toripalimab)	55	TPS/TMB		ORR	33.3%	3.0%	Wang 2019 (5)
					PFS	2.7 months	1.9 months	
					OS	12.1 months	4 months	

GC, gastric cancer; CPS, combined positive score; TPS, tumor proportion score; AGC, advanced gastric cancer; MSI, microsatellite instability; mCRC, metastatic colorectal cancer; ORR, objective response rate; CRR, complete response rate; DCR, disease control rate; PFS, progression-free survival; OS, overall survival; PFS, progression-free survival; HCC, hepatocellular carcinoma; TMB, tumor mutation burden.

Subclonal genotypes, transcriptome, and epigenetic changes may influence immune escape and explain intratumoral PD-L1 diversity. In MSS mCRC patients, PD-L1 mutations were shown to mediate immune escape of a subset of tumor subclones against avelumab, thereby affecting the efficacy of immunotherapy in a subset of patients expressing high-affinity FcγR3a (45). Patients with PD-L1 mutated subclones showed a higher-than average therapeutic benefit, showing slow dynamics reversing on avelumab withdrawal, which suggest that PD-L1 mutations may mediate the development of resistance to the direct antitumor effects of avelumab. Further trials are required to evaluate the specific clinical benefits of immunotherapy in this subset of MSS mCRC patients.

PD-L1 is a strong predictor of the efficacy of cancer immunotherapy, although it is not completely perfect. There is considerable heterogeneity in the expression of PD-L1 between tumors and within tumors, and its expression is not homogenous even in a pair of independent tumor lesions (46). Paolino found that PD-L1 expression was underestimated in biopsy compared with resected specimens, while the positive expression of PD-L1 was higher in metastatic lymph nodes than in primary tumors (47). In addition, the expression of PD-L1 is inducible and dynamic, and may be affected by interferons and toll-like receptor ligands, radiotherapy, targeted therapy, and chemotherapy (48, 49). Currently, PD-L1 expression is mainly detected using immunohistochemical methods, and there is considerable variability with respect to the positive thresholds set by different studies, the antibody detection platforms and the detection techniques used. Recent studies have analyzed assays for evaluating PD-L1 expression, and the results demonstrated that SP263 assay tended to increase the degree of positive expression while SP142 assay tended to stain better immune cells (50). Cerbelli explored the inter-observer reliability and correlation between PD-L1 expression assays, which revealed high agreement between the 22C3 PharmDx assay and the SP263 assay, suggesting that the two antibodies are interchangeability in immunotherapy (51). Furthermore, the lack of predictive utilization of PD-L1 expression is also attributable to dynamic changes in the TME and the fact that baseline tests may not reflect rapid variation of PD-L1 expression due to adaptive responses to treatment (52). These limitations further heighten concerns about the accuracy of PD-L1 expression. Therefore, formulation of a standardized PD-1/PD-L1 detection method is a key imperative. The expression of PD-L1 alone is not enough to screen people who can fully benefit from ICI treatment.

Moreover, amplification or higher expression of PD-L1 showed an independent association with dismal survival in HCC patients, authenticating the PD-1/PD-L1 axis as rational immunotherapeutic targets for HCC. Since the dynamics and turnover of translation and transcription levels may be different, clinical biomarker assessments usually adopt the PD-L1 protein level rather than the mRNA level for HCC (53). However, PD-L1 expression failed to predict the efficacy of ICI therapy in MSS CRC (10, 11). In a recent study, PD-L1 mRNA levels, but not the protein level, was associated with CD8+ T cell infiltration and better prognosis of immunotherapy for MSS CRC. The inconsistency between PD-L1 protein and mRNA expressions may indicate that PD-L1 regulation occurs post transcriptionally (42). Furthermore, some studies have shown a strong correlation of PD-L1 mRNA expression with prognosis in the context of NSCLC and malignant melanoma (54). The predictive value of PD-L1 or PD-L1 mRNA for immunotherapy is likely to vary due to cancer heterogeneity. Larger prospective clinical trials are required to verify whether PD-L1 mRNA can be a potential predictor of response to immunotherapy in patients with digestive system cancers. Moreover, there is a need to explore models that combine multiple biomarkers and/or assay methods to improve the predictive ability for immunotherapy efficacy.

### 2.3 Blood Biomarkers

#### 2.3.1 Peripheral Blood Biomarkers

Peripheral blood biomarkers in routine clinical practice can help predict the treatment outcomes of digestive system cancers, and thereby facilitate risk-stratification and therapeutic decision-making for this patient population. Among the common laboratory tests, neutrophil-to-lymphocyte ratio (NLR) and absolute lymphocyte count (ALC) may be effective surrogate markers for predicting the outcome of immunotherapy for digestive system cancers. Namikawa found that the NLR after 4 weeks of nivolumab therapy in the CR or partial response (PR) group was significantly lower than that in the stable disease (SD) or progression disease (PD) group (2.2 vs 2.9, *P*=0.044) (55). Besides, another study of 26 advanced GC patients treated with nivolumab also investigated on the role of NLR before the first cycle (NLR_pre_) and NLR at two weeks after the first administration (NLR_post_) in predicting the efficacy of immune therapy (56). After stratifying patients into high NLR (≥5) and low NLR (<5) groups, the mPFS was shorter in the high NLRpre arm (45 vs 87 days) and high NLRpost arm (28 vs 94 days). Consistently, high NLR_pre_ arm (175 vs 290 days) and high NLR_post_ arm (69 vs 290 days) showed significantly shorter OS. In the study by Ohta et al., the 6-month OS rate of patients with ALC>1,600/mL (100%) and NLR<4 (63%) was greater than that of patients with ALC<1600 mL (35%) and patients with NLR>4 (33%), respectively (57). The potential reason of dynamic changes in NLR as a predictive indicator may be related to changes in the relative proportion of circulating lymphocytes during nivolumab therapy. As a specific biomarker of HCC, alpha-foetoprotein (AFP) may also help predict the efficacy of immunotherapy. Patients with early response (decrease in AFP level by at least 20% from pre-treatment level within the initial 4 weeks of treatment) exhibited longer OS (28.0 vs 11.2 months) and PFS (15.2 vs 2.7 months), becoming an independent predictor of longer OS (58).

Some compound blood biomarkers also have ability to predict clinical efficacy. The association of such a cost-effective and widely accessible biomarker like NLR to TMB seems an implementable strategy worth exploring. Valero performed a retrospective cohort study of 1714 patients with various cancer types (including GC, CRC, HCC, PDAC, and ESCA) who were treated with ICI therapy; the probability of benefit from ICI was found to be significantly higher in the NLR low/TMB-H group compared to the NLR high/TMB-L group (62). Composite markers, such as prognostic nutrition index (PNI) and Glasgow prognostic score (GPS), which are based on a combination of routine blood parameters (serum albumin, lymphocyte count, C-reactive protein, and hypoalbuminemia), have been used to evaluate inflammatory status in GC patients. In a study, pre-treatment PNI in the CR or PR group was significantly better than that in the SD or PD group (37.1 vs 32.1, respectively; *P*=0.011). PNI at 8 weeks post-treatment and pre-treatment GPS showed significant association with poor efficacy of nivolumab therapy (55).

However, the methodology used to determine the cutoff levels of these peripheral blood biomarkers is unclear in many studies. Owing to different cutoff levels used in previous studies, further studies are required to determine the optimal cut-off levels of hematological markers for different cancers. In other cancers such as NSCLC or melanoma, the levels of lactate dehydrogenase, carcinoembryonic antigen, carbohydrate antigen199, and carbohydrate antigen125 have also been shown to be related to efficacy of immune therapy (63). Recent studies suggest that peripheral blood LAG-3 protein may be an important biomarker for predicting ICI efficacy in patients with melanoma (64). However, further research is required to explore accurate hematological markers in the context of digestive system cancers.

#### 2.3.2 Liquid Biopsy Biomarkers

Long-term monitoring of the occurrence and development of cancers is an important aspect of precision medicine. However, tissue biopsy is not convenient enough for this purpose. Therefore, use of liquid biopsy in the context of cancer treatment is a useful evolving trend. Circulating tumor cells (CTCs), circulating tumor DNA (ctDNA), and exosomes together constitute the three major goals of liquid biopsy.

ctDNA mutation load can not only predict the response before treatment, but the change of ctDNA immediately after treatment can also strongly predict the response to immunotherapy. The ORR of patients with higher ctDNA mutation load was significantly greater than that of lower ctDNA (83% vs 7.7%, *P*=0.0014), and was shown to improve mPFS (87 days vs not reached). Besides, all patients who showed increasing ctDNA after treatment experienced PD within 100 days, and demonstrated significant decrease in DCR (92% vs 25%) and ORR (58% vs 0%), resulting in a shortened mPFS (123 days vs 66 days) (59). Moreover, Bratman found that the decline in ctDNA levels after pembrolizumab therapy was an independent predictor in patients with solid tumors. They also found that assessment of the changes in ctDNA levels in combination with the evaluation criteria for solid tumors in the third cycle of treatment could help identify patients who are unlikely to benefit from ICI treatment at an early stage. All 12 patients with ctDNA clearance during ICI therapy survived with median follow-up of 25 months (65). Furthermore, the proportion of CTCs with high PD-L1 expression at baseline and monitoring the early dynamic changes in CTC were found to predict the clinical efficacy of Sintilimab (61). At baseline, patients above the cutoff value of PD-L1^high^ CTCs had significantly longer mPFS compared with those below the value (4.27 vs 2.07 months, *P*=0.002). At 9 weeks after the initiation of therapy, patients with PD-L1^high^ CTCs <2 showed significantly better mPFS than patients with PD-L1^high^ CTCs ≥2 (3.4 vs 2.1 months, *P*=0.031). The ratio of PD-L1^high^ is of great value in predicting the efficacy of ICI therapy, and the results of a prospective study of this predictive marker have shown promising results. It is crucial to obtain enough high-purity CTC cells to determine the expression of PD-L1 protein.

Plasma cell free DNA (cfDNA) is a degraded DNA fragment released into plasma. Yang et al. constructed a copy number variations (CNV) risk score model based on peripheral blood cfDNA to predict the efficacy of ICI-based therapy in patients with hepatobiliary cancers. In cohorts receiving combination of ICI-based therapies, patients with lower CNV risk scores had longer OS [not reported (NR) vs 6.5 months] and PFS (6.17 vs 2.6 months) than those with high CNV risk scores (see **Table 3**) (60).

**Table 3:** Predictive ability of hematological biomarkers for response to ICI therapy for digestive system cancers.

Type of predictors	Cancer type	ICI therapy	Number	Outcome	Beneficial outcome	Adverse outcome	Reference
ALC NLR	Advanced GC	anti-PD-1 (Nivolumab)	15	6-month OS rate	ALC>1,600/mL: 100%	ALC<1600 mL: 35%	Ohta 2020 (57)
					NLR<4: 63%	NLR>4: 33%	
NLR	Advanced GC	anti-PD-1 (Nivolumab)	26	mPFS	Low NLR_pre_ (≥5): 87 days	High NLR_pre_ (<5): 45 days	Ogata T 2018 (56)
					Low NLR_post_ (≥5): 94 days	High NLR_post_ (<5): 28 days	
				mOS	high NLR_pre_ (≥5): 290 days	low NLR_pre_ (<5): 175 days	
					high NLR_post_ (≥5): 290 days	low NLR_post_ (<5): 69 days	
AFP	Advanced HCC	anti-PD-1/PD-L/ CTLA-4	60	OS	Early AFP response: 28	early AFP nonresponders: 11.2	Shao 2019 (58)
				PFS	Early AFP response: 15.2	early AFP nonresponders: 2.7	
ctDNA	Metastatic GC	anti-PD-1 (Pembrolizumab)	61	ORR	the upper tertile of ctDNA mutational load: 83%	the lower two tertiles of ctDNA mutational load: 7.7%	Kim 2018 (59)
					decreasing ctDNA: 58%	increasing ctDNA: 0%	
				mPFS	the lower two tertiles of ctDNA mutational load: not reached	the upper tertile of ctDNA mutational load: 87 days	
					decreasing ctDNA: 123 days	increasing ctDNA: 66 days	
				DCR	decreasing ctDNA: 92%	increasing ctDNA: 25%	
cfDNA	Hepatobiliary cancers	anti-PD-1	108	mOS	lower CNV risk scores: not reached	Higher CNV risk scores:6.5 months	Yang 2021 (60)
				mPFS	lower CNV risk scores: 6.17 months	Higher CNV risk scores:2.6months	
CTCs	Advanced solid tumor	anti-PD-1 (Sintilimab)	34	mPFS	High levels of PD-L1^high^CTCs before therapy: 4.27 months	Low levels of PD-L1^high^CTCs before therapy: 2.07 months	Yue 2018 (61)
					PD-L1^high^CTCs post therapy<2: 3.4 months	PD-L1^high^CTCs post therapy≥2: 2.1 months	

ALC, absolute lymphocyte count; NLR, neutrophil-to-lymphocyte ratio; AFP, alpha-foetoprotein; ctDNA, circulating tumor DNA; CTCs, circulating tumor cells; cfDNA, cell free DNA; GC, gastric cancer; HCC, hepatocellular carcinoma; PFS, progression-free survival; mOS, median overall survival; OS, overall survival; mPFS, median progression-free survival; DCR, disease control rate.

The above studies demonstrate the potential clinical utility of blood-based surveillance in patients receiving ICI therapy. Blood is a specimen that can be provided at the time of diagnosis. Compared with a single-point biopsy, blood tests are not easily interfered by sampling bias, which reduces the heterogeneity associated with sampling of tumor tissue. In addition, blood tests are minimally invasive, allow repeated sampling, and are associated with better patient compliance. Therefore, future basic and clinical research is required to further clarify the predictive value of soluble PD-L1 and bTMB in digestive system cancers (66). Furthermore, soluble B7-CD28 family inhibitory immune checkpoint proteins (including soluble CTLA-4, soluble B7-1 and soluble B7-H4) play a wide role in anti-cancer immunomodulatory regulation. Therefore, these may serve as valuable prognostic biomarkers that can predict the therapeutic response, while also opening up new opportunities for anticancer immunotherapy (67).

### 2.4 Tumor Microenvironment (TME)

The TME is a complex tumor ecosystem that supports tumor growth and metastatic dissemination (68), and can reflect the response of the tumor to immunotherapy. TILs status is an important component of the heterogeneity of TME and mediates adaptive immunity.

A comprehensive immuno-genomic analysis of tumor microenvironment immunetypes (TMITs) classified it into four subgroups based on the expressions of PD-1 and CD8 in TILs. In the category of digestive system cancers, TIMT I subgroup (PD-L1 immunoreactivity of tumor cells and CD8 high expression of TILs) showed a significantly higher number of mutations or neoantigens in CRC and GC patients receiving anti- PD-1/PD-L1 therapy (69). On survival analysis according to TMIT, TMIT I showed the most prominent favorable prognostic effect. Furthermore, Noh performed another TMIT study in patients with small intestinal adenocarcinoma. TMIT I subgroup (PD-L1-positive tumor cells and CD8-high TILs) and TMIT III subgroup (PD-L1-positive tumor cells and CD8-low TILs) showed the best and worst outcomes of immunotherapy, respectively (73). However, the classification standards for immune type are not completely standardized, and further characterization of the immune type of patients with digestive system cancers may provide a breakthrough for ICI therapy in these patients.

Some studies indicated that tumors with high expression of T cell-"inflamed" phenotype show favorable response to immunotherapy. T-cell inflammatory gene expression profile (GEP) can be used as an inflammatory marker of T-cell inflamed TME, which was associated with longer PFS and higher ORR in patients treated with pembrolizumab (33). Tumors with "inflammatory" T cell infiltration are characterized by activation of type I IFN, immune potentiating chemokines that attract T cells, antigen presentation, and CD8^+^ T cells, while tumor tissues with "non-inflammatory" T cell infiltration lack such expression and activation. The researchers hypothesized that a treatment regimen that converts non-inflammatory T-cell subtypes to inflammatory T-cell subtypes may enhance the sensitivity of the tumor to treatment regimens that depend on T-cell activity, thereby enhancing the therapeutic efficacy of immunotherapy (74). Additional analysis showed that combined use of TMB and GEP stratified Pan-tumor into groups that showed different clinical responses to pembrolizumab monotherapy, with both response rate and PFS strongest in groups with GEP high and TMB high (75). TMB and GEP independently predict the therapeutic response owing to their unique characteristics of capturing neoantigenicity and T cell activation to provide new patterns for predicting response to immunotherapy.

The tumor microbiome is composed of tumor type-specific intracellular bacteria, which is an important part of the TME. Different types of tumors have their own unique microbiota. Proteobacteria dominate the microbiota of pancreatic cancer. Bacteria can be found in CD45+ immune cells, which indicates that they may affect or reflect the immune status of the TME. Researchers have found differences in the differential abundance of microbes in immunotherapy responders and non-responders. Some microorganisms are related to the effectiveness of ICI therapy, which may be related to the metabolic ability of bacteria in the TME (76).

Immune score has attracted increasing clinical attention, which is determined using standardized operating procedures and specialized image-analysis software to quantify the density of CD3+ and CD8+ T cells in the tumor and its invasive edge. The 5-year recurrence risk of CRC patients with high immune scores (8%) was significantly lower than that of patients with lower than medium (19%) and low immune score (32%) (see **Table 4**) (70).

**Table 4:** Predictive ability of EBV, TME, BMI, and BLN for response to ICI therapy for digestive system cancers.

Type of predictors		Cancer type	ICI therapy	Number	Outcome	Beneficial outcome	Adverse outcome	Reference
EBV		Metastatic GC	anti-PD-1 (Pembrolizumab)	6	PR	EBV positive: 100%	–	Kim 2018 (62)
					mDOR	EBV positive: 8.5	–	
TME	TMIT	Small intestinal adenocarcinoma	anti-PD-1/PD-L1	195	mOS	TMIT Type I: 146.6 months	TMIT Type III: 12.1 months	NOH 2018 (69)
	Immunoscore	CRC	anti-PD-1/PD-L1/ CTLA-4	2,681	Risk of recurrence at 5 years	High immunoscore: 8%	Low immunoscore:32%	Pagès 2018 (74)
BMI		Advanced multiple cancers	anti-PD-1/PD-L1	976		**BMI≥25**	**BMI<25**	Cortellini 2019 (93)
					OS	26.6 months	6.6 months	
					PFS	11.7 months	3.7 months	
					TTF	9.3 months	3.6 months	
BLN		Refractory AGC	anti-PD-1	58		**BLN^low^ TMB-H**	**BLN^high^ TMB-L**	Wei 2021 (99)
					mPFS	Not reached	1.7 months	
					mOS	Not reached	2.7 months	
					ORR	37.5%	0%	
					DCR	62.5%	13.3%	

EBV, Epstein-barr virus; GC, gastric cancer; PR, partial response; PFS, progression-free survival; mOS, median overall survival; OS, overall survival; mPFS, median progression-free survival; TME, tumor micro-environment; TMIT, tumor microenvironment immunetypes; ICI, immune checkpoint inhibitors; CRC, colorectal cancer; BMI, body mass index; TTF, time to progression; ORR, objective response rate; DCR, disease control rate; TMB, tumor mutation burden; BLN, baseline lesion number; AGC, advanced gastric cancer; mDOR, median duration of response.

The TILs status and neoantigen changes after chemoradiotherapy can partly reflect the dynamic changes in the TME, which may induce immunotherapy response in patients who were hitherto non-responsive. How to screen such patients, or how to change the TME to improve response to immunotherapy, is an important direction for immunotherapy research to expand the application of immunotherapy and improve the effectiveness of immunotherapy.

### 2.5 Immune-Related Adverse Events

ICI treatment interferes with normal immune tolerance and triggers immune activation in normal tissues, leading to various irAEs. Of note, irAEs often indicate a good response to ICI treatment. In a multi-center retrospective study of patients with metastatic or unresectable GI cancer, patients with irAEs had longer mPFS (not reached vs 3.9 months) and mOS (not reached vs 7.4 months) than patients without irAEs (77). The above research highlighted the predictive potential of irAEs as a clinical biomarker in this population. In the studies of nivolumab for GC, a study of 65 GC patients showed significant improvement in mPFS (7.5 vs 1.4 months) and mOS (16.8 vs 3.2 months) in patients with irAEs compared with those without irAEs (78). Further study of 29 GC patients also confirmed the predictive effect of irAEs on PFS, with a significant increase in the median PFS in the irAEs group (5.8 vs 1.2 months) (55). Reactive causal capillary endothelial proliferation (RCCEP) is an extremely common skin irAE caused by camrelizumab. In a study, the mPFS (3.2 vs 1.9 months) and mOS (17.0 vs 5.8 months) of HCC patients with RCCEP was significantly greater than those of patients without RCCEP (79). In addition to HCC, camrelizumab therapy also increased the mOS of patients with RCCEP in a study of 228 patients with ESCC (10.1 vs 2.5 months) (see **Table 5**) (80).

**Table 5:** Predictive performance of irAES for response to ICI therapy for digestive system cancers.

Cancer type	ICI therapy	Number	Outcome	irAES	None-irAES	Reference
GI cancer	anti-PD-1	76	mPFS	Not reached	3.9 months	Das 2020 (77)
			mOS	Not reached	7.4 months	
Advanced GC	anti-PD-1 (Nivolumab)	65	mPFS	7.5 months	1.4 months	Masuda 2019 (78)
			mOS	16.8 months	3.2 months	
Advanced GC	anti-PD-1 (Nivolumab)	29	mOS	5.8 months	1.2 months	Namikawa 2020 (55)
HCC	anti-PD-1 (Camrelizumab)	217	mPFS	3.2 months	1.9 months	Wang 2020 (79)
			mOS	17.0 months	5.8 months	
ESCC	anti-PD-1 (Camrelizumab)	228	mOS	10.1 months	2.5 months	Huang 2020 (80)

GI, gastrointestinal; GC, gastric cancer; HCC, hepatocellular carcinoma; mPFS, median progression-free survival; mOS, median overall survival; ESCC, esophageal squamous cell carcinoma; irAES, immune-related adverse events.

The relationship between irAEs and ICI efficacy is potentially attributable to presence of similar antigens in tumor cells and other normal tissues (81). When the immune system is activated, it targets not only tumor cells but also non-tumor sites. Furthermore, infiltration of CD4+ and CD8+T cells in the damaged sites leads to auto-immune dysfunction, resulting in a series of clinical adverse reactions. However, not all irAEs indicate better efficacy of immunotherapy, because severe irAEs, such as immune-associated pneumonia and myocarditis, can be fatal. Therefore, dynamic management of irAEs should still be strictly cautious, following the five principles of prevent, anticipate, detect, treat and monitor.

### 2.6 Patient-Related Biomarkers

#### 2.6.1 Sex

In a meta-analysis of 20 randomized trials of immunotherapy for multiple tumors, including GC, survival benefits of ICI therapy differed significantly between men and women [pooled OS hazard ratio (HR): 0.86 vs 0.72, *P*=0.0019]. Although the differences were statistically significant, due caution should be exercised before drawing any definitive conclusions. Future research should focus on improving treatment outcomes in women and exploring different immunotherapy regimens for men and women. New immunotherapy studies should be designed to ensure that more women are enrolled in trials to obtain a more comprehensive evaluation (82).

#### 2.6.2 Gut Microbiome Biomarkers

The dynamic balance of gut microbiome plays a positive role in maintaining the homeostasis of the immune system. However, disruption of this balance may participate in the occurrence and development of malignant tumors by regulating host immune regulation, apoptosis, autophagy, and other pathways. A recent study showed that gut microbiome can influence cancer immune reference point and it is thought to induce specific memory T cells by interferon-γ secreting CD4+and CD8+ T cells, which are associated with favorable results of anti-tumor immunotherapy (83).

Fluckiger identified major histocompatibility complex (MHC) class I-binding epitopes in the tail length tape measure protein (TMP) of a prophage found in the bacteriophage Enterococcus hirae. Mice carrying Enterococcus hirae containing this prophage mounted CD8+ T cell response upon anti-PD-1 immunotherapy, and improved the therapeutic effect of ICI therapy (83). In clinical studies, the response of patients to immunotherapy was shown to be related to the abundance of gut microbiome. Fecal samples from HCC patients responding to ICI therapy showed higher taxa richness than those of non-responders. At the 6th week of treatment, there was a significant difference in the beta diversity of the gut microbiome. In non-responders, Proteobacteria became predominant at week 12, while Akkermansia muciniphila and Ruminococcaceae, were significantly increased in ICI responders (84). Mager found that Bifidobacterium pseudolongum (B.pseudolongum), Lactobacillus johnsonii, and Olsenella species can enhance the efficacy of ICI therapy in CRC mouse models, and B. pseudolongum was found to enhance the response to immunotherapy through increased systemic translocation of inosine and activated anti-tumor T cells (85). Moreover, Drewes’s study indicated that the efficacy of the anti-PD-L1 and anti-CTLA-4 in CRC patients is reliant on commensal bacteria, such as bifidobacteria and bacteroides (86). During the development of pancreatic cancer, tumor local immunity and gut microbiome interact with tumor and change synergistically with histopathological progression. Combined targeting of gut microbiome composition and metabolic pathways may effectively improve the efficacy of immunotherapy in PDAC (87).

These studies provide fascinating insights into the relationship between gut microbiome and antitumor efficacy of ICI therapy, suggesting that the gut microbiome can be used as a predictor of immunotherapy efficacy, and even become a potential treatment modifier. In the studies confirming the efficacy of intestinal microflora in regulating immunotherapy, different microflora were found to play a major role, which may be related to the tumor species and the patient population. Further studies should be conducted to explore the mechanism by which gut microbiome activates the TME (88). Given the biological and clinicopathologic heterogeneity among different tumors and individuals, the prospective value of gut microbiome biomarkers needs to be further explored in a larger cohort with a unitary tumor type.

#### 2.6.3 Epstein-Barr Virus Biomarkers

Epstein-Barr virus associated gastric carcinoma (EBVaGC) has unique molecular biology characteristics, and is, therefore, considered as an independent gastric cancer subtype in studies. An estimated 8%–10% of GCs are associated with EBV infection, and patients with EBVaGC were found to have a better prognosis compared with other genotypes (89). With the ongoing advances in the field of precision medicine, especially in the field of immunotherapy, EBVaGC diagnosis and ICI therapy have become contemporary research hotspots. Follow-up studies have shown that EBVaGC shows greater sensitivity to ICI therapy. In a study by Panda et al., compared with MSI tumors, EBVaGC subtypes had a lower mutation burden, but showed stronger evidence of immune infiltration; in addition, RNA-SEQ data showed high expressions of immune checkpoint pathway (PD-1, CTLA-4 pathway) genes, which seemed to confer greater benefit of avelumab treatment against EBVaGC subtypes (90). Furthermore, in another study, the efficacy of pembrolizumab in GC patients was associated with positive EBV. All EBVaGC patients achieved PR with a median duration of response of 8.5 months and the ORR was 100% (59).

The above studies indicated that EBV may serve as a predictor of the efficacy of immunotherapy for GC, while the following studies provide a strong rationale for testing of PD-1 blockade in EBV-positive GC. EBVaGC is characterized by marked intratumoral or peritumoral infiltration of immune cells. The infiltrating lymphocytes are mainly CD8+ T cells, and large infiltration of CD8+T cells in GC tissues is often accompanied by high expression of PD-L1 (91). In addition, the IL-12 mediated signal intensity in EBV-positive tumors suggests the presence of a robust immune cell response, which provides a basis for the detection of ICI in EBVaGC (92). Besides, Derks reported enrichment of interferon-γ driven gene signature in EBVaGC and the amplification of PD-L1 in EBVaGC cells was significantly greater than that in other GC subtypes, which also implies that EBVaGC may show greater sensitivity to PD-1/PD-L1 immunotherapy (93). The 2020 NCCN guidelines for GC suggested that tumor EBV status may be a biomarker for precision therapy of GC. Accurate detection is the premise of precise treatment, and EBV examination is crucial for ICI therapy and prognosis of EBVaGC patients.

#### 2.6.4 Body Mass Index

Obesity is a global social and public health problem. Increased body mass index (BMI) is a known health hazard and is associated with an increased risk of cancer (94). A 5 kg/m^2^ increase in BMI showed a strong association with oesophageal, colon, and gallbladder adenocarcinoma. However, recent studies have highlighted the potential role of obesity as a biomarker of the efficacy of cancer immunotherapy. Cortellini conducted a retrospective study of advanced cancer patients consecutively treated with anti-PD-1/PD-L1 inhibitors. They found that mPFS, mOS, and time to treatment failure were significantly longer for patients with BMI≥25 kg/m^2^ (71).

The potential underlying mechanism by which obesity modulates the response to ICI therapy may involve obesity-induced immune aging and PD-1-mediated T cell dysfunction by leptin. PD-1-mediated T cell dysfunction was shown to enhance the efficacy of immune checkpoint blockade and confer long-term survival benefits (95). The relationship between BMI and OS should be interpreted with caution, as it may potentially be confounded by methodological limitations and heterogeneity with respect to study design. Therefore, it is vital to consider that patients with the same BMI may have significantly different body compositions and different prognosis, which reflect that BMI is not an adequate indicator of regional obesity. In addition to BMI, other indices such as waist circumference, visceral fat mass, subcutaneous fat mass, total body fat percentage, and trunk fat percentage should be integrated into comprehensive indicators to explore their relationship with benefits of ICI therapy (96). At present, the mechanism of “obesity paradox” needs to be further explored.

#### 2.6.5 Nonalcoholic Steatohepatitis (NASH)

There are several etiological factors for HCC, among which NASH is an important driver of HCC. A recent study found that NASH limits the immunotherapy response of HCC to anti-tumor surveillance, and is a predictor of unfavorable outcomes of ICI therapy. After anti-PD1 treatment in NASH-related HCC mice, pre-existing HCC tissue showed no regression and there was an increase in CD8+/PD1+T cells in HCC tissue, suggesting that the activated immune cells may not play an immune surveillance role, but show the potential of tissue destruction (97). To further determine the clinical significance of disrupted immune surveillance in NASH after ICI treatment, Pfister conducted a meta-analysis of the three large randomized controlled trials (CHECKMATE-459, IMbrave150, and KEYNOTE-240) of patients with advanced HCC and found that non- alcoholic fatty liver disease (NAFLD) was associated with shorter mOS (5.4 vs 11.0 months) and mPFS (8.8 vs 17.7 months). After adjusting for potential confounders, NAFLD still showed an independent association with shorter survival in HCC patients treated with ICI. Due to the limited size of the study cohort, future prospective clinical studies are required to provide more robust evidence.

#### 2.6.6 *Helicobacter pylori*

*Helicobacter pylori* (H. pylori) colonizes the gastric mucosa of 50% of the world's population and is related to geographical factors such as diet and lifestyle. H. pylori actively manipulates host tissue, establishes an immunosuppressive environment, and mediates immune regulation, which negatively affects a large number of immune cell types associated with antitumor immunity. Therefore, H. pylori infection is considered to attenuate the response to cancer immunotherapy (98). Oster's team used an MC38 colorectal adenoma tumor model to evaluate whether H. pylori reduces the efficacy of anti-CTLA-4 therapy. In this study, the tumor size of mice that were not infected with H. pylori was significantly smaller than that of the infected mice. Furthermore, they evaluated the effect of H. pylori infection on the immunotherapy efficacy in tumors developing *in situ* using a model of azoxymethane/dextran sodium sulfate colon cancer. Notably, the number of colon tumors in uninfected mice treated with anti-CTLA-4 was significantly lower than that in H. pylori infected mice. This study provided evidence that the presence of H. pylori in the gastric microbiota may jeopardize the efficacy of immunotherapies (99).

#### 2.6.7 Other Predictive Markers

Patients with high initial tumor burden often have poor immune status and show poor response to immunotherapy. Recent research suggested that baseline lesion number (BLN) is a potential indicator of tumor burden, which takes priority over tumor size and can reflect the effect of tumor biology on immunotherapy sensitivity; in addition, BLN can be used in combination with TMB for better stratification of patients with respect to the risk of immunotherapy. The BLN-Low group showed better ORR and DCR compared with BLN-High group (15.4% vs 5.3% and 86.96% vs 54.29%, respectively). However, combined use of BLN and TMB showed better efficacy in predicting the benefits of immunotherapy. This study suggested that use of a combination of clinical and molecular biomarkers may have greater clinical relevance. The BLN-Low (≤ 5) and TMB-H (≥12 mutations/Mb) groups showed higher ORR (37.5% vs 0%) and DCR (62.5% vs 13.3%) , as well as longer PFS (not reached vs 1.7 months) and OS (not reached vs 2.7 months), compared with the BLN-High and TMB-L groups (see **Table 4**) (72).

## 3 Conclusion

In an era of immunotherapy, several biomarkers have been identified to predict the response to immunotherapy. However, the vast majority of biomarkers have shown limited predictive value in the context of digestive system cancers. This may be attributable to the fact that digestive system cancers is a large class of tumors with considerable heterogeneity, and which is possibly itself involved in regulation of gut microbiome and the TME. This may also be related to the lower response rate of digestive system tumors to immunotherapy compared with NSCLC and melanoma. At present, MSI is mainly used to predict the effectiveness of immunotherapy for digestive system cancers; however, owing to the low overall incidence of MSI-H in the digestive system, it has limited clinical application. There is a need to explore novel biomarkers in the context of digestive system cancers.

The conflicting conclusions pertaining to the predictive efficacy of biomarkers in previous studies may be due to the differences with respect to tumor types, treatment methods, and detection standards used. A single biomarker is unlikely to accurately predict the effect of ICI treatment, and is liable to be affected by dynamic changes in the immunogenicity of the tumor and TME. Therefore, in the future, artificial intelligence and big data research can be used to build multidimensional and multi-variable predictive models. Through large-sample analysis, multi-platform dynamic detection before and after treatment can be conducted, so as to obtain comprehensive predictive markers with the best predictive performance to guide clinical treatment. Timothy Chan team performed a comprehensive analysis of multiple biological factors (TMB, copy number change score, HLA-1 evolutionary difference, HLA-1 loss of heterozygosity, MSI, BMI, gender, NLR, tumor stage, immunotherapy drugs, age, tumor type, chemotherapy before immunotherapy, and blood indicators such as albumin, platelets and hemoglobin) (100) . The machine learning model (named RF16) was shown to predict the efficacy of ICI therapy with a high sensitivity and specificity, spanning 1479 patients with 16 cancer types (including CRC). The analysis demonstrated that TMB has the greatest influence on the immunotherapy efficacy, followed by the history of chemotherapy. However, the impact of MSI does not seem to be large, and researchers believe that this is most likely due to the very strong correlation between MSI and TMB. The prediction consistency index of RF16 was higher than that of TMB, and the response to immunotherapy predicted by RF16 was also significantly associated with longer OS.

Previous research has focused on sensitive markers to identify subsets of patients who are more likely to benefit from immunotherapy. In recent years, increasing attention has been paid to immunotherapy resistance genes and super-progressive genes. Exploration of new biomarkers should take into account both the sensitive and super-progressive directions. Further in-depth research on predictive biomarkers of immunotherapy will help leverage the full potential of ICI therapy in the realm of personalized medicine for cancer. Targeting different individuals, cancer types, and immune status, as well as TME-related data, such as molecular characteristics, microbial composition, T cell receptor library diversity, tumor-related gene mutations or drug resistance mutations, may help improve the model’s predictive ability. Immunotherapy prediction models, especially for digestive system cancers, should be validated and improved using a larger and more representative patient population in the future. Improvement in the predictive models will help inform treatment strategies for digestive system cancers and open new vistas for individualized immunotherapy.

## Author Contributions

Data acquisition and data analysis were performed by JTW, JW, ZM, and XM. YM, and BC prepared the figure and tables. The first draft of the manuscript was written by JTW and JW, and the manuscript was further commented and approved by all authors. All authors contributed to the article and approved the submitted version.

## Funding

This study was supported by the National Natural Science Foundation of People’s Republic of China [grant number 82173056]; the Digestive Medical Coordinated Development Center of Beijing Hospitals Authority. No: XXT01; Beijing key clinical specialty (2018-2020); The pilot project of clinical collaboration with traditional Chinese medicine and western medicine in major refractory disease-Esophageal cancer (2019-ZX-005).

## Conflict of Interest

The authors declare that the research was conducted in the absence of any commercial or financial relationships that could be construed as a potential conflict of interest.

## Publisher’s Note

All claims expressed in this article are solely those of the authors and do not necessarily represent those of their affiliated organizations, or those of the publisher, the editors and the reviewers. Any product that may be evaluated in this article, or claim that may be made by its manufacturer, is not guaranteed or endorsed by the publisher.
